# High Hepcidin Levels Promote Abnormal Iron Metabolism and Ferroptosis in Chronic Atrophic Gastritis

**DOI:** 10.3390/biomedicines11092338

**Published:** 2023-08-22

**Authors:** Yashuo Zhao, Jianing Zhao, Hongyu Ma, Yan Han, Weichao Xu, Jie Wang, Yanru Cai, Xuemei Jia, Qingzhong Jia, Qian Yang

**Affiliations:** 1 The First Affiliated Hospital, Hebei University of Chinese Medicine, Shijiazhuang 050013, China; 2 Department of Gastroenterology, Hebei Province Hospital of Chinese Medicine, Shijiazhuang 050013, China; 3 Hebei Technology Innovation Center of TCM Combined Hydrogen Medicine, Hebei University of Chinese Medicine, Shijiazhuang 050200, China; 4 School of Pharmacy, Hebei Medical University, Shijiazhuang 050017, China

**Keywords:** hepcidin, chronic atrophic gastritis, ferroptosis, iron, IL-6/STAT3 signaling pathway

## Abstract

Background: Chronic atrophic gastritis (CAG) is a chronic inflammatory disease and premalignant lesion of gastric cancer. As an antimicrobial peptide, hepcidin can maintain iron metabolic balance and is susceptible to inflammation. Objectives: The objective of this study was to clarify whether hepcidin is involved in abnormal iron metabolism and ferroptosis during CAG pathogenesis. Methods: Non-atrophic gastritis (NAG) and chronic atrophic gastritis (CAG) patient pathology slides were collected, and related protein expression was detected by immunohistochemical staining. The CAG rat model was established using MNNG combined with an irregular diet. Results: CAG patients and rats exhibited iron deposition in gastric tissue. CAG-induced ferroptosis in the stomach was characterized by decreased GPX4 and FTH levels and increased 4-HNE levels. Hepcidin, which is mainly located in parietal cells, was elevated in CAG gastric tissue. The high gastric level of hepcidin inhibited iron absorption in the duodenum by decreasing the protein expression of DMT1 and FPN1. In addition, the IL-6/STAT3 signaling pathway induced hepcidin production in gastric tissue. Conclusion: Our results showed that the high level of gastric hepcidin induced ferroptosis in the stomach but also inhibited iron absorption in the intestines. Inhibiting hepcidin might be a new strategy for the prevention of CAG in the future.

## 1. Introduction

Chronic atrophic gastritis (CAG) is a prevalent and challenging chronic digestive disease. As the world’s fifth most common malignant tumor, gastric cancer (GC) is the most frequently occurring malignant tumor of the digestive system [1,2]. The progression from a precancerous state to GC is a protracted process. In 1975, Correa proposed an “inflammatory–cancer transformation” model, which remains widely accepted today. This model outlines the sequence of events as follows: normal gastric mucosa, chronic inflammation, CAG, intestinal metaplasia, dysplasia, and ultimately GC. Among these, CAG and intestinal metaplasia are the most significant precancerous gastric lesions [3,4]. Hence, it is imperative to employ the approach of obstructing or reversing the advancement of CAG towards the premalignant lesions of GC [5].

CAG is characterized by the degeneration of glandular structures, exposure of vessels, and collapse of the reticular skeleton within the gastric mucosa, which can be evaluated via endoscopic, histological, and serological methods [6]. Patients diagnosed with CAG commonly experience symptoms, such as upper abdominal pain, indigestion, indigestion, bloating, nausea, vomiting, belching, loss of appetite, and weight loss [6,7]. The progression of CAG might lead to diminished gastric acidity and ascorbic acid levels, potentially causing iron deficiency or, in severe cases, iron deficiency anemia [8,9].

Iron is an essential metal for all biological activities and is mainly absorbed in the intestine. Dietary Fe^3+^ is converted to Fe^2+^ by duodenal cytochrome b (Dcyt b) and subsequently absorbed by divalent metal transporter 1 (DMT1) located in the apical membrane of duodenal epithelial cells [10]. The absorbed iron is either stored in ferritin or released into the bloodstream by ferroportin 1 (FPN1) in the basement membrane and then transported to various target organs [11]. Hepcidin plays a critical role in maintaining iron balance and is predominantly produced and released in the liver [12]. Hepcidin can bind to FPN1, the sole iron-exporting protein, causing the degradation of FPN1, thereby regulating iron metabolism through the control of iron absorption in the small intestine and iron release in macrophages [12]. Furthermore, an elevated expression of hepcidin has been found to impede the release of iron from cells, resulting in the accumulation of iron in localized areas. Excessive iron accumulation can further exacerbate reactive oxygen species (ROS) generation and oxidative stress damage through the Fenton reaction [13].

As hepcidin is an antimicrobial polypeptide, its expression is often influenced by inflammation and iron status [10]. Clinical studies have found iron deficiency and high levels of hepcidin in the serum in patients with Helicobacter pylori-infected nodular gastritis [14,15]. Furthermore, animal studies demonstrated that hepcidin was also expressed in the base/body of the glandular stomach and primarily expressed and secreted by gastric parietal cells [3,8]. In a CAG animal model, the elevated expression of hepcidin in gastric juice had an unfavorable effect on the absorption of iron in the duodenum, or even could potentially result in the development of peptic ulcers [8]. Therefore, the maintenance of the dynamic balance of hepcidin plays a constructive role in the progression of the “inflammation–cancer transformation” of CAG.

Ferroptosis is a form of cell death caused by the excessive accumulation of iron-dependent lipid free radicals [16]. Free iron ions (mainly Fe^2+^) in the cell interact with hydrogen peroxide via the Fenton reaction, which leads to the lipid peroxidation of polyunsaturated fatty acids (PUFAs) of biofilms, which is the fundamental molecular mechanism known to initiate ferroptosis [16,17]. Glutathione peroxidase 4 (GPX4) is a key regulatory factor of ferroptosis, and it can remove phosphatide peroxide precisely and efficiently, thus inhibiting the occurrence of ferroptosis [17,18]. Previous research has demonstrated that chronically elevated iron levels and ferroptosis contribute to tumor development [16]. However, the impact of iron levels and ferroptosis on atrophic gastritis remains uncertain.

Therefore, the objective of this investigation was to clarify the potential involvement of hepcidin in the pathogenesis of CAG-induced ferroptosis. This objective was pursued by examining both CAG patients and animal models. Consequently, this study provides innovative theoretical foundations for the effective clinical management of CAG.

## 2. Materials and Methods

### 2.1. Reagents

Reagents: potassium ferrocyanide (Sigma-Aldrich, Saint Louis, MO, USA, 13425), RIPA lysis buffer (Servicebio, Wuhan, China, G2002), a protease inhibitor (Thermo Scientific, Waltham, MA, USA, A32955), and 1-Methyl-3-nitro-1-nitrosoguanidine (MNNG, Meilunbio, Dalian, China, MB0455).

Antibodies: 4-hydroxynonenal (4-HNE, Arigo Biolaboratories, Hsinchu, Taiwan, ARG70025), GPX4 (Huabio, Woburn, MA, USA, ER1803-15), H^+^-K^+^-ATPase (GeneTex, Irvine, CA, USA, GTX22866), hepcidin (Affinity Biosciences, Cincinnati, OH, USA, #DF6492), H-ferritin (FTH, Abcam, Cambridge, MA, USA, ab183781), Interleukin-6 (IL-6, Immunoway, Plano, TX, USA, YT0470), Tumor necrosis factor-α (TNF-α, Affinity Biosciences, Cincinnati, OH, USA, AF7014), Interleukin-1β (IL-1β, Affinity Biosciences, Cincinnati, OH, USA, AF5103), Transducer and activator of transcription 3 (STAT3, Cell Signaling Technology, Danvers, MA, USA, #4904), p-STAT3 (Cell Signaling Technology, #9145), DMT1 (Absin, Tokyo, Japan, abs112967), FPN1 (Alpha Diagnostic Intl., San Antonio, TX, USA, MTPP11-S), β-Tubulin (Servicebio, Wuhan, China, GB12139), β-actin (Cwbiotech, Beijing, China, CW0096), and glyceraldehyde-3-phosphate dehydrogenase (GAPDH, Servicebio, Wuhan, China, GB15004).

### 2.2. Patients and Samples

This study involved a total of 38 samples obtained from patients diagnosed with CAG or non-atrophic gastritis (NAG) in the gastric antrum at least one year before their inclusion in the study. All specimens were acquired through biopsy and diagnosed using gastroscopy and pathological analysis. The pathological findings were collected during the patient’s initial visit, with any treatment. The baseline information regarding the patients is shown in Table 1. This study was approved by the Ethics Committee of Hebei Province Hospital of Chinese Medicine (Approval number: HBZY2020-KY-042-01) and was performed following the Helsinki II Declaration.

### 2.3. Animal Experiment

Male Wistar rats (SPF grade, 150 ± 20 g) were purchased from Beijing Vital River Laboratory Animal Technology Co., Ltd. (Beijing, China). Upon arrival, all the rats were transferred to the Experimental Animal Centre and acclimated to the environment for seven days before the commencement of the experiments. The experimental procedures involving the animals adhered to the National Institutes of Health Guide for the Care and Use of Laboratory Animals and were granted approval by the Animal Care and Use Committee of Medical Ethics of the Hebei University of Chinese Medicine (Approval number DWLL2019022).

The rats were subjected to random allocation into two groups: the control (Con) group and the CAG model group (n = 10 for each group). Animal models of CAG were induced using oncogenic agents (primarily MNNG) and saturated sodium salt (to simulate high salt intake) [19,20]. The rats in the CAG group were administered MNNG (180 µg/mL) in drinking water combined with an irregular diet: two days of full feeding and one day of fasting with intragastric administration of 2% sodium salicylate (1 mL/100 g), which was repeated for 24 weeks.

### 2.4. Histopathological Examinations

H&E staining was used to observe the basic structure of stomach tissue and was performed according to a standard protocol. Stomach paraffin sections (5 µm) were dewaxed and rehydrated with xylene and gradient alcohol. The sections were immersed in hematoxylin and differentiated with hydrochloric-acid ethanol. After being counterstained with eosin, the sections were dehydrated with gradient alcohol, cleared in xylene, and mounted.

### 2.5. Transmission Electron Microscopy

Mitochondrial ultrastructure was observed using a transmission electron microscope (TEM). After anesthesia, fresh stomach tissue was quickly removed, cut into 1 mm^3^ blocks, and soaked in glutaraldehyde. The blocks were dehydrated and embedded in Araldite. Then, the blocks were cut into ultrathin sections and stained with uranyl acetate and lead citrate. Finally, images were observed under an electron microscope (H-7650, Hitachi, Tokyo, Japan).

### 2.6. Perls’ Staining

Perls’ staining was used to detect the Fe^3+^ levels and distribution in the stomach and duodenum. After dewaxing and rehydration, the paraffin sections were incubated with 3% hydrogen peroxide. Then, the sections were soaked in fresh 1% Perls’ dye solution containing potassium ferrocyanide and hydrochloric acid. After 10 h in the dark, the sections were rinsed with PBS and then enhanced by DAB staining. Finally, imaging was performed on a DM300 (Leica) microscope. The mean density of Fe was calculated by IPP 6.0 software.

### 2.7. Immunohistochemistry

The dewaxing-free sections were incubated with 3% hydrogen peroxide to block endogenous peroxidase. Citric acid solution (10 mM, pH 6.0) was heated using a microwave to repair the antigen. The sections were incubated with goat serum at 37 °C for 60 min.

Immunohistochemistry was performed as follows: The sections were incubated with primary antibodies against GPX4, 4-HNE, hepcidin, FTH, IL-6, TNF-α, IL-1β, and p-STAT3 at 4 °C for 12 h. After being washed with PBS, the sections were incubated with the HRP-conjugated secondary antibody at 37 °C for 40 min. After DAB-enhanced staining, counterstaining with hematoxylin was performed to label the cell nucleus.

Double immunofluorescence was performed as follows: The sections were incubated with a mixture of rabbit anti-hepcidin and mouse H^+^-K^+^-ATPase overnight at 4 °C. Then, a secondary antibody mixture of goat anti-rabbit DL-549 (red) and goat anti-mouse DL-488 IgG (green) was added and incubated for 60 min at 37 °C. Finally, the sections were sealed with an anti-fluorescence quencher and imaged via a fluorescence microscope (DM6B, Leica, Germany).

### 2.8. Western Blotting

Frozen stomach tissues were homogenized and centrifuged, and the supernatants were collected. A BCA protein assay kit was used to quantify the total protein concentrations. SDS‒PAGE was used to separate the denatured proteins, which were then transferred to PVDF membranes using a wet transblot system (Bio-Rad, Hercules, CA, USA). At room temperature, the blots were blocked with 5% non-fat dry milk for 2 h. Primary antibodies against GPX4, 4-HNE, IL-6, FTH, p-STAT3, STAT3, GAPDH, β-actin, and β-tubulin were added and incubated with the blots overnight at 4 °C. Then, the blots were incubated with HRP-conjugated secondary antibodies at room temperature for 1 h. The immunoreactive protein blots were imaged using ECL chemiluminescence. ImageJ software (version 1.8.0, Rawak Software Inc., Stuttgart, Germany) was used to analyze the mean grey values of the target bands.

### 2.9. Statistical Analyses

The results are presented as the mean ± SEM. The statistical analysis was performed using SPSS 23.0 software with a *t*-test, and *p* < 0.05 indicated a significant difference. Prism 9.0 software (GraphPad Software, Inc., La Jolla, CA, USA) was used.

## 3. Results

### 3.1. Results

#### 3.1.1. Ferroptosis Was Involved in CAG Injury

**Figure 1:**
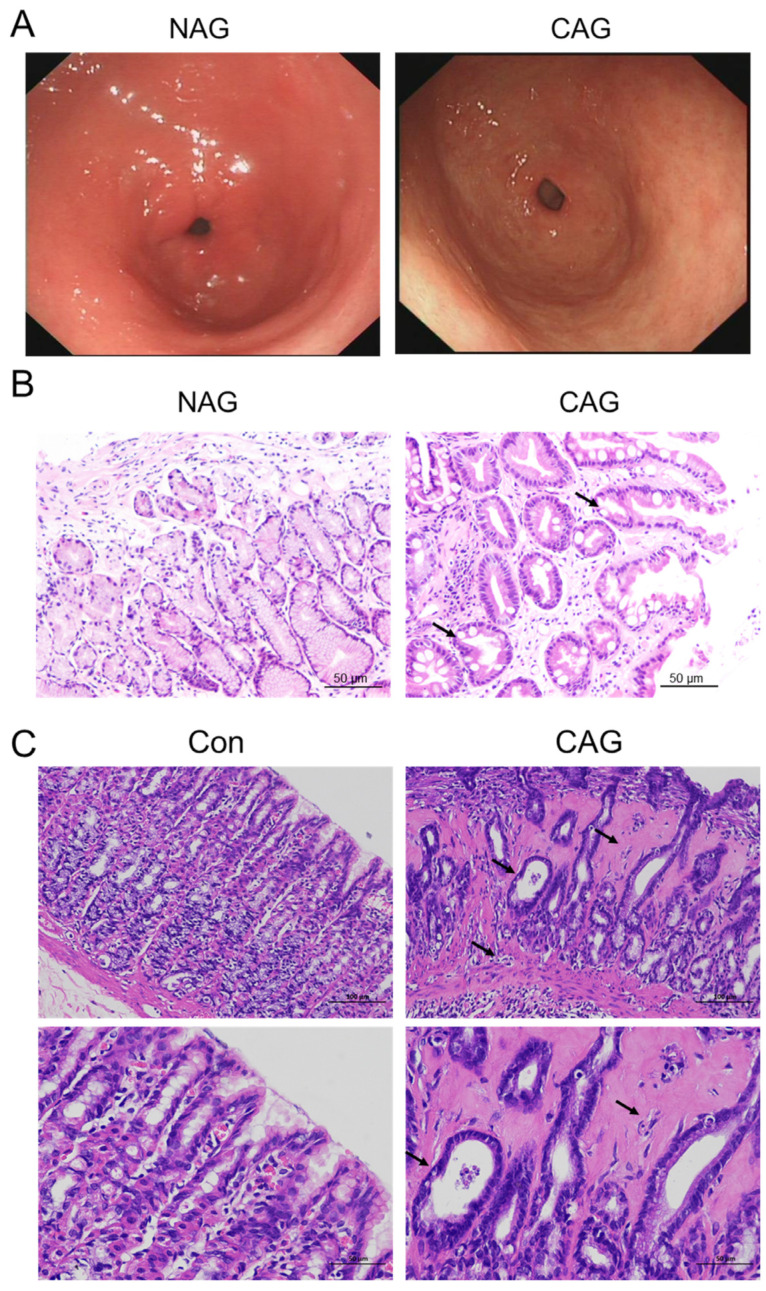
Histopathological evaluation of CAG patients and rat models. (**A**) Endoscopic analysis of NAG and CAG patients (n = 5). (**B**) HE staining of NAG and CAG patient samples (Scale bar = 50 μm, n = 5). (**C**) HE staining of normal and CAG rat samples (Scale bar = 100 or 50 μm, n = 5). The arrows indicated local atrophy of the mucosal layer, reduced number of gastric glands, and hyperplasia of connective tissue. NAG: non-atrophic gastritis patients; CAG: chronic atrophic gastritis patients or rats; Con: the control group of rats.

The histological examination of biopsy samples obtained during upper digestive endoscopy is widely regarded as the definitive method for CAG or NAG [3]. In this study, we specifically selected pathological sections from patients diagnosed with mild to moderate NAG and CAG in the antrum. As shown in Figure 1A, the endoscopic findings demonstrated a rough and uneven gastric antrum mucosa with the presence of multiple white spots, along with a faintly visible submucosal vascular network. The histological examination of HE staining in CAG patients revealed significant local atrophy in the mucosal layer, a reduced count of gastric glands, hyperplasia of connective tissue, and a limited number of dilated gastric glands (Figure 1B,C).

**Figure 2:**
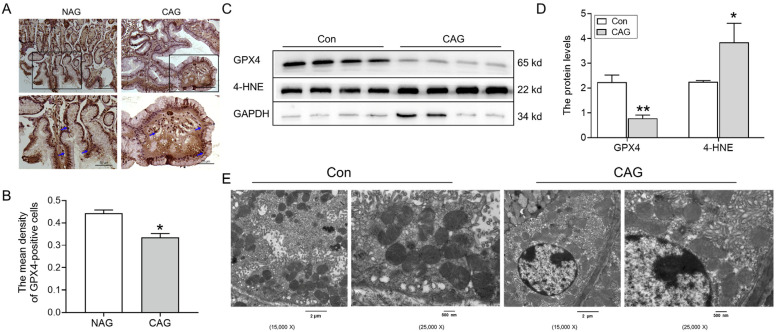
Ferroptosis was involved in CAG injury. (**A**) GPX4 immunofluorescence staining in the gastric tissue of patients (Scale bar = 100 or 50 μm, n = 3). The bottom images were the larger images of the frame in the top images, and the arrows indicate the location of GPX4-positive cells. (**B**) The mean density of GPX4-positive cells in Panel (**A**). (**C**, **D**) The expression and analysis of GPX4 and 4-HNE protein levels in the gastric tissue of rats, as determined by Western blotting (n = 4). (**E**) TEM analysis of the gastric tissue of rats with 15,000 or 25,000 magnification (n = 3). The data are presented as the mean ± SEM. * *p* < 0.05, ** *p* < 0.01 vs. NAG or Con groups. NAG: non-atrophic gastritis patients; CAG: chronic atrophic gastritis patients or rats; Con: the control group of rats.

To examine whether ferroptosis was involved in the pathogenesis of CAG-related injury, we initially assessed the expression of GPX4 in both CAG patients and animal models. The immunohistochemical analysis demonstrated a significant decrease in GPX4 levels in patients with CAG compared to those with NAG (Figure 2A,B). Additionally, Western blotting analysis revealed a decrease in GPX4 levels in the gastric tissue of the CAG animal group (Figure 2C). Furthermore, the levels of 4-HNE were found to be upregulated in the gastric tissue of the CAG animal group (Figure 2D). Moreover, the ultrastructure of mitochondria was examined, and TEM revealed that mitochondria were decreased in size and had fragmented ridges in the CAG group compared to the control group (Figure 2E). These findings collectively suggested the occurrence of ferroptosis in the stomach during CAG.

#### 3.1.2. An Excess of Iron Was Observed in the Gastric Tissue of CAG

**Figure 3:**
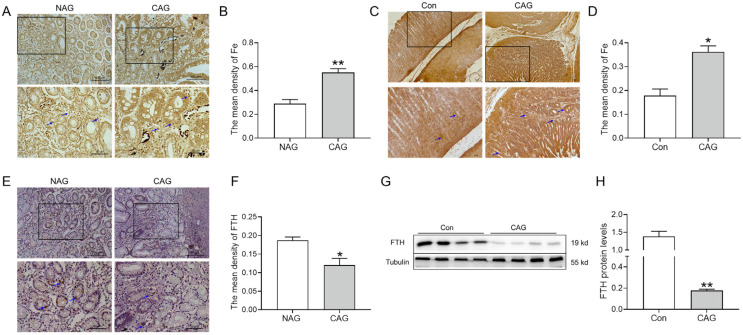
Iron levels in CAG gastric tissue. (**A**) Perls’ staining of the gastric tissue of patients (n = 3). (Scale bar = 100 or 50 μm, n = 3). (**B**) The mean density of Fe in Panel (**A**). (**C**) Perls’ staining of the gastric tissue of rats (Scale bar = 25 μm, n = 3). (**D**) The mean density of Fe in Panel (**C**). (**E**) FTH immunofluorescence staining in the gastric tissue of patients (Scale bar = 100 or 50 μm, n = 3). (**F**) The mean density of FTH-positive cells in Panel (**E**). (**G**,**H**) The expression and analysis of FTH protein levels in the gastric tissue of rats, as determined by Western blotting (n = 4). The bottom images were the larger images of the frame in the top images, and the arrows indicate the location of Fe or FTH-positive cells as shown in (**A**,**C**,**E**). The data are presented as the mean ± SEM. * *p* < 0.05, ** *p* < 0.01 vs. NAG or Con groups. NAG: non-atrophic gastritis patients; CAG: chronic atrophic gastritis patients or rats; Con: the control group of rats.

Ferroptosis consistently exhibits a correlation with iron overload. Therefore, we elevated iron levels in the gastric tissue of CAG. Perls’ staining and analysis demonstrated a notable accumulation of iron in the gastric tissue of CAG patients (Figure 3A,B). Likewise, the same results were obtained in CAG rats (Figure 3C,D). Additionally, a decline in FTH levels was observed in the gastric tissue of CAG patients by immunohistochemical staining (Figure 3E,F). Western blot results also revealed that FTH protein levels were decreased in the gastric tissue of CAG rats (Figure 3G,H), indicating impaired iron storage capacity. These findings cooperatively indicated the presence of iron deposition in the gastric tissue of CAG.

#### 3.1.3. Upregulation of Hepcidin in Gastric Tissue of CAG

**Figure 4:**
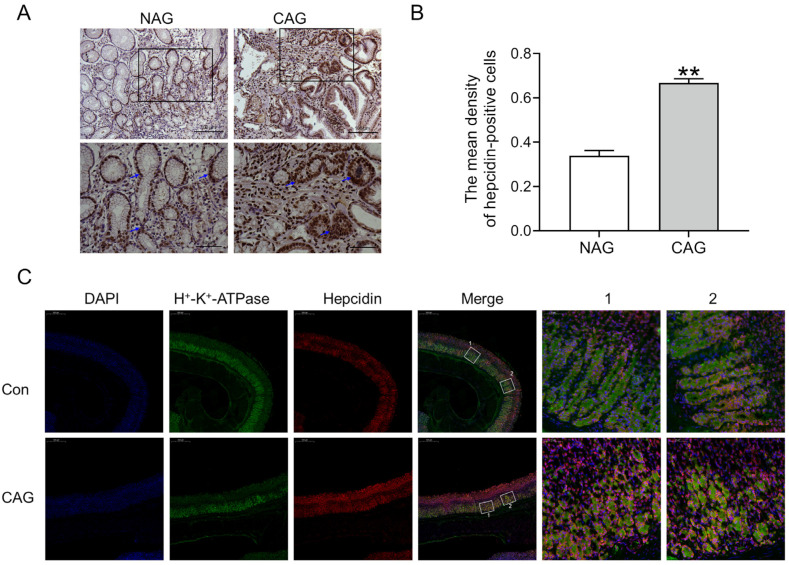
Hepcidin levels were elevated in CAG gastric tissue. (**A**) Hepcidin immunofluorescence staining in the gastric tissue of patients (Scale bar = 100 or 50 μm, n = 3). The bottom images were the larger images of the frame in the top images, and the arrows indicate the location of hepcidin-positive cells. (**B**) The mean density of hepcidin-positive cells in Panel (**A**). (**C**) Double immunofluorescence labeling of H^+^-K^+^-ATPase (green color) and hepcidin (red color) in the gastric tissue of CAG rats (Scale bar = 500 or 50 μm, n = 3). The right images (marked 1, 2) were the larger images of the frame in the Merge images. The data are presented as the mean ± SEM. ** *p* < 0.01 vs. NAG or Con groups. NAG: non-atrophic gastritis patients; CAG: chronic atrophic gastritis patients or rats; Con: the control group of rats.

We further evaluated whether iron deposition was associated with abnormal hepcidin expression. The immunohistochemical results showed that hepcidin protein levels were dramatically increased in the gastric tissue of CAG patients compared to those in NAG patients (Figure 4A,B). In the stomach, hepcidin is mainly expressed and released by parietal cells, and H^+^-K^+^-ATPase serves as a specific marker for parietal cells. Double immunofluorescence labeling demonstrated that hepcidin (red color) was increased while parietal cells (green color) were shrunken in the atrophic position of gastric tissue in CAG rats (Figure 4C). These findings provide evidence of significant induction of hepcidin during the development of CAG.

#### 3.1.4. IL-6/STAT3 Signaling Pathways Were Activated by CAG

**Figure 5:**
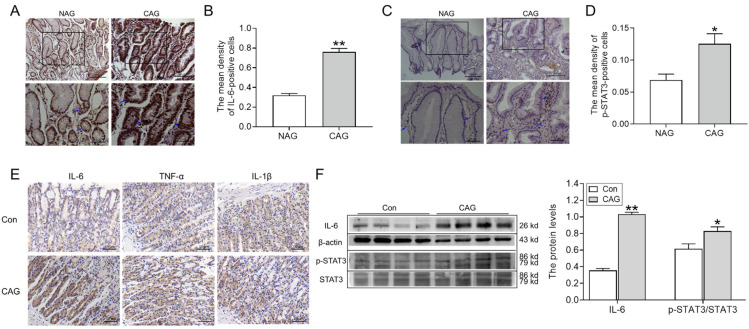
The IL-6/STAT3 signaling pathways were activated in CAG gastric tissue. (**A**) IL-6 immunofluorescence staining in the gastric tissue of patients (Scale bar = 100 or 50 μm, n = 3). (**B**) The mean density of IL-6-positive cells in Panel (**A**). (**C**) p-STAT3 immunofluorescence staining of the gastric tissue of patients (Scale bar = 100 or 50 μm, n = 3). (**D**) The mean density of p-STAT3-positive cells in Panel (**C**). The bottom images were the larger images of the frame in the top images, and the arrows indicate the location of IL-6-positive cells (**A**) or p-STAT3-positive cells (**C**). (**E**) IL-6, TNF-α, and IL-1β immunofluorescence staining in the gastric tissue of CAG rats (n = 3). (**F**) The expression of IL-6 and the ratio of p-STAT3/STAT3 in the gastric tissue of CAG rats, as determined by Western blotting (n = 4). The data are presented as the mean ± SEM. * *p* < 0.05, ** *p* < 0.01 vs. NAG or Con groups. NAG: non-atrophic gastritis patients; CAG: chronic atrophic gastritis patients or rats; Con: the control group of rats.

The expression of hepcidin is responsive to inflammation, thus prompting us to investigate the expression of inflammatory cytokines and associated signaling pathways in the gastric region. As shown in Figure 5A,B, the expression of IL-6-positive cells significantly increased in the gastric tissue of patients with CAG, compared to NAG. At the same time, p-STAT3 protein levels within the gastric tissue of patients with CAG were also elevated, especially in areas where inflammation was obvious (Figure 5C,D). Immunohistochemical analysis further demonstrated IL-6-, TNF-α-, and IL-1β-positive cells all increased in the gastric tissue of CAG rats when compared to the rats in the Con group (Figure 5E). Additionally, Western blotting analysis confirmed IL-6 levels and the ratio of p-STAT3/STAT3 (Figure 5F) were markedly increased in gastric tissue of the CAG rat model compared to normal rats (Figure 5F). All in all, these observations provided compelling evidence for the heightened presence of IL-6/STAT3 signaling pathways that participated in hepcidin expression during CAG.

#### 3.1.5. Abnormal Iron in the Duodenum of CAG Rats

**Figure 6:**
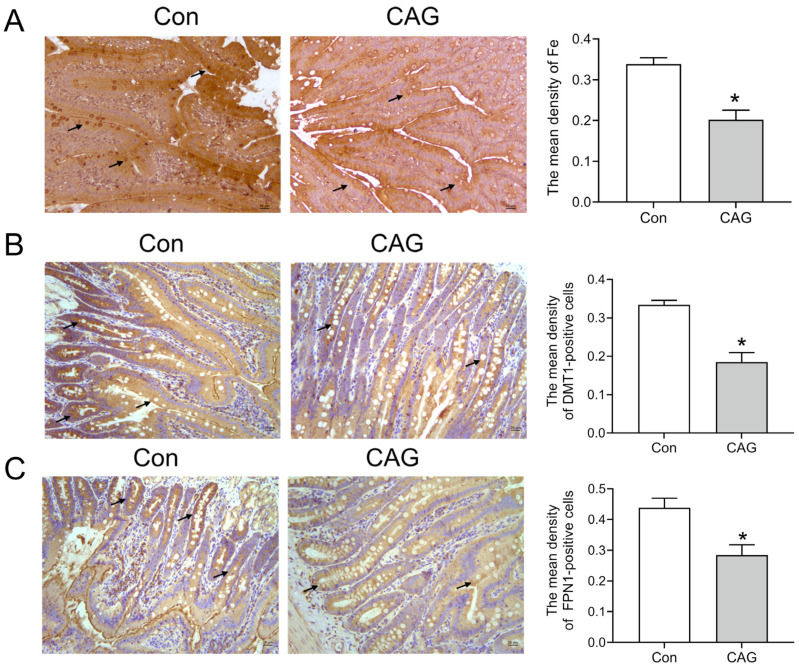
Iron levels and iron-related transport proteins in the duodenum of CAG rats. (**A**) Perls’ staining and analysis of Fe levels in the duodenum of CAG rats. (**B**) DMT1 immunofluorescence staining and analysis of the duodenum of CAG rats. (**C**) FPN1 immunofluorescence staining and analysis of the duodenum of CAG rats. Scale bar = 25 μm, and the arrows indicate the location of Fe, DMT1-positive cells, or FPN1-positive cells. The data are presented as the mean ± SEM, n = 3. * *p* < 0.05 vs. Con group. Con: Control group of rats. CAG: chronic atrophic gastritis of rats.

Hepcidin can regulate the release of iron from tissue stores and the absorption of dietary iron from the intestines. Perls’ staining showed that Fe levels in the duodenum were decreased in the CAG group compared to those in the control group (Figure 6A). Cells positive for DMT1 (the main iron absorption protein) were decreased in the intestines of the CAG group (Figure 6B). Moreover, cells positive for FPN1 (the main iron exportin protein) were significantly reduced in the intestines of the CAG group (Figure 6C). These results showed that lower levels of DMT1 and FPN1 contributed to inadequate iron uptake in the intestines during CAG.

## 4. Discussion

Previous research has demonstrated that CAG patients always exhibited aberrant iron metabolism or iron deficiency anemia, yet the specific molecular mechanism remains largely elusive. In this study, we effectively procured pathological sections from CAG patients and established a CAG rat model [21], thereby revealing an underappreciated role of hepcidin in CAG. Specifically, our findings indicated that elevated levels of hepcidin contributed to the occurrence of ferroptosis in the stomach and impaired iron absorption in the intestines.

Normally, the presence of free Fe^2+^ in the intracellular space, referred to as the labile iron pool (LIP), is minimal. When there is an excess of iron, it accumulates not only within the intracellular space but also in the stromal tissue (Figure 3A,B). The majority of intracellular iron is stored in ferritin, and ferritin degradation leads to an increase in LIP levels, promoting lipid peroxidation or ferroptosis [22,23]. FTH, a subtype of the iron storage protein ferritin, plays a crucial role in maintaining iron balance by binding to iron ions. Additionally, FTH exhibits antioxidant properties due to its ferrous oxidase activity [24]. In cancer cells, intracellular iron addiction facilitates various processes including cellular proliferation, invasion, migration, and drug metabolism. Previous research showed that FTH, in conjunction with p53, played a tumor-suppressive role [24]. Our findings indicate a significant decline in FTH levels in both CAG patients and rats with gastric tissue (Figure 3E–H), suggesting that it may be a crucial factor in the transition from inflammation to cancer during the progression of gastric cancer.

Ferroptosis is a distinct form of cell death that relies on iron-dependent lipid peroxidation, distinguishing it from apoptosis, autophagy, and necrosis [16]. Ferroptosis has been observed in both inflammatory bowel disease (IBD) and GC [25,26]. The antitumor effects of ferroptosis have been demonstrated, but it can also promote tumor growth and survival [27]. GPX4 serves as a crucial regulator of ferroptosis, capable of detecting and converting large and detrimental lipid hydroperoxides into harmless lipid alcohols through the utilization of reduced glutathione [4,16]. 4-HNE, an indicator of lipid peroxidation products, is elevated and coincides with the occurrence of ferroptosis [17,28]. Mitochondria, known as the primary sites of ROS production, play a crucial role in various cellular processes, including apoptosis, autophagy, and ferroptosis [29]. The higher levels of 4-HNE, lower levels of GPX4, and abnormal structure of mitochondria in gastric tissue suggested the occurrence of ferroptosis in CAG (Figure 2). Ferritinophagy, a selective autophagy process facilitated by nuclear receptor coactivator 4 (NCOA4), leads to the degradation of FTH in lysosomes, resulting in the release of iron to the LIP. This subsequently triggers the Fenton reaction, ROS production, and ultimately ferroptosis [24]. As the central iron regulator, hepcidin can cause the ubiquitination of FPN1, leading to an excessive accumulation of iron in the cell. Previous research has shown that elevated levels of hepcidin could promote ferroptosis in cases of cerebral ischemia–reperfusion [28] and subarachnoid hemorrhage [30]. In our study, the observed decrease in FTH (Figure 3) and concurrent increase in hepcidin levels (Figure 4) contributed to the deposition of iron and ferroptosis in the gastric tissue of CAG.

A growing number of studies have focused primarily on the impact of Helicobacter pylori infection on hepcidin levels in the intestinal tract [2,4,31]. This infection is known to be a significant risk factor for the development of CAG and GC, leading to anemia and depletion of serum iron stores, inducing anemia and depleting serum iron storage levels [32,33]. Moreover, it was observed that Helicobacter pylori infection was resistant to iron supplementation [34]. Notably, higher levels of hepcidin mRNA and protein have been detected in gastric tumor tissues of GC patients [35]. Subsequent investigations have revealed that hepcidin is primarily localized and secreted by gastric parietal cells, with subsequent release into the gastric lumen rather than the bloodstream [8,35].

**Figure 7:**
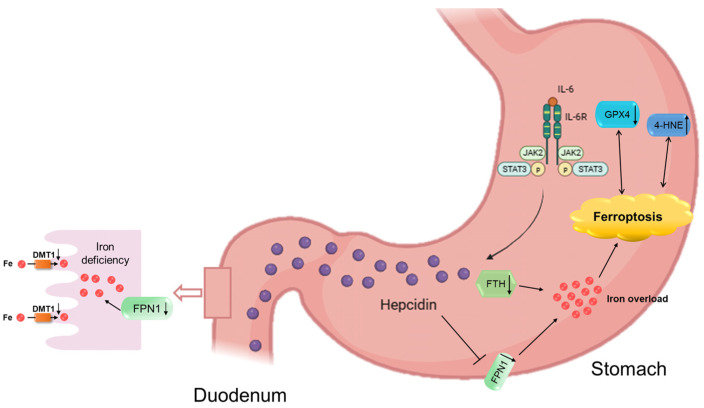
A schematic showing hepcidin involvement in CAG injury. CAG can increase IL-6 expression and then activate hepcidin via the JAK2/STAT3 signaling pathway. On the one hand, high levels of hepcidin restrain FPN1, causing iron overload and ferroptosis in CAG gastric tissue, as indicated by a higher level of 4-HNE and a lower level of GPX4. On the other hand, hepcidin is expressed and released by parietal cells and flows into the duodenum. Subsequently, high levels of hepcidin inhibit FPN1 expression in the basal membrane of epithelial cells and DMT1 expression in the brush border membrane of the duodenum, resulting in iron uptake deficiency in the duodenum.

Multiple studies have reported a notable increase in gastric hepcidin levels and an increase in serum hepcidin/pro-hepcidin levels among patients with Helicobacter pylori infection [8,33]. This infection has been found to elevate both total iron and hepcidin levels in AGS cells, thereby disrupting intracellular iron balance [8,36]. Notably, the eradication of Helicobacter pylori did not result in any changes to serum hepcidin levels, suggesting that hepcidin primarily exerts localized rather than systemic effects in gastric tissue [33]. It is known that iron absorption is reliant on an acidic gastric environment with a low pH, which aids in stabilizing Fe^2+^ [32]. The expression of hepcidin, which can regulate acid secretion, has been shown to have a significant protective role, but it may also contribute to the development of gastric ulcers through excessive acid production [8]. Research has provided evidence indicating that hepcidin can impede the absorption of iron in the intestines by facilitating the degradation of FPN1 [12] and DMT1 [8,37]. Correspondingly, we observed an upregulation of hepcidin and a downregulation of DMT1 and FPN1 in CAG rats induced by MNNG in conjunction with an irregular diet (Figure 6 and Figure 7). Therefore, abnormal gastric acid might be an unfavorable factor for intestinal iron absorption or even iron deficiency anemia in CAG patients.

It is widely considered that the inducible expression of hepcidin is predominately controlled at the transcriptional level, which is significantly enhanced by various inflammatory stimuli. Hepcidin, which is an acute-phase reactant, was proven to be upregulated via IL-6 during bacterial infection or inflammation [33]. The initial evidence of the association between hepcidin and lipopolysaccharide (LPS) was demonstrated in hepatocytes [38]. Secreted IL-6 binds to the IL-6 receptor (IL-6R) and then initiates the activation of Janus kinase 2 (JAK2) and subsequently STAT3 phosphorylation. This activation leads to the stimulation of hepcidin synthesis through the binding of a STAT3 response element in the hepcidin promoter [39,40]. IL-6 was highly expressed and secreted from both gastric epithelial cells and inflammatory cells infiltrating the gastric mucosa [41] in CAG patients regardless of Helicobacter pylori infection status [42]. A study has demonstrated a parallel induction of IL-6 expression and Helicobacter pylori infection in AGS cells [8]. Inhibiting IL-6 and inactivating STAT3 could ameliorate H. pylori-associated CAG and prevent gastric carcinogenesis [43]. Moreover, the expression levels of IL-6, IL-6R, and STAT3 proteins were all increased in the gastric tissue of GC patients [35]. IL-6 was also found to play a crucial role in activating target genes associated with differentiation, survival, apoptosis, and proliferation, thereby influencing the process of gastric carcinogenesis [35]. Therefore, the inhibition of the IL-6/STAT3 signaling pathway not only diminishes hepcidin expression but also mitigates the likelihood of transitioning to GC. Wang et al. demonstrated that Cyclovirobuxine D inhibited L-type calcium currents, thus suppressing LPS-induced IL-6/STAT3/hepcidin signaling pathway expression and ferroptosis [44]. It is undeniable that some ion channel control by hepcidin may be involved in CAG.

In the stomach, inflammation leads to damage to the gastric glands and facilitates the progression of potential complications. Previous research has indicated that CAG patients with intestinal metaplasia exhibited significantly increased numbers of intramucosal macrophages, and these are more likely to develop into GC [45]. During Helicobacter pylori infection, macrophages are recruited to the gastric mucosa and transform into the M1 type, which releases pro-inflammatory cytokines, such as T helper 1 (Th1), interferon gamma (IFN-γ), TNF-α, and IL-1β. These pro-inflammatory factors further contribute to the inflammatory response [46]. The immune function of macrophages is influenced by their ability to regulate cellular iron homeostasis [47], and M1 macrophages have a high ferritin content and are susceptible to iron deposition [48]. The hepcidin level is related to the immune infiltration level of macrophages, positively correlating with the M1 type and negatively correlating with the M2 type [49]. A study demonstrated that activated hepcidin led to iron overload in macrophages within plaques, resulting in cellular cholesterol imbalance and the formation of foam cells [50]. Additionally, cigarette tar promoted the progression of atherosclerosis by inducing ferroptosis in macrophages through the NF-κB-activated hepcidin/FPN1/SLC7A11 pathway [51]. Normally, hepcidin is localized and secreted by gastric parietal cells, and Helicobacter pylori infection can induce hepcidin expression in lymphocytes present in the gastric mucosal lymphoid follicles [52]. Our study also verified that some inflammatory cytokines, such as TNF-α, IL-6, and IL-1β, were increased in the serum of CAG rats induced by MNNG combined with an irregular diet [53]. Consequently, maintaining the expression of hepcidin in macrophages has a positive significance for the prevention of CAG-related damage.

There were still some limitations in this study. First, we focused on the hepcidin in gastric tissue of CAG patients; however, we did not distinguish the Helicobacter pylori infection status in the enrolled patients. This limitation might cause some deviation in the final results of the study. Additionally, we focused primarily on the expression levels of proteins rather than the specific cell types responsible for their expression. Therefore, further investigation is warranted to elucidate the role of hepcidin in CAG without Helicobacter pylori infection, as well as to explore potential pharmacological interventions aimed at impeding the transition from inflammation to carcinogenesis.

## 5. Conclusions

In this paper, we systematically studied hepcidin, iron status, and ferroptosis using gastric tissue in CAG patients and animal models with MNNG combined with an irregular diet. In conclusion, we found that hepcidin-mediated abnormal iron and ferroptosis were involved in CAG-induced injury. Notably, the elevated levels of hepcidin induced ferroptosis specifically in the stomach, and they impeded iron absorption in the intestines. Furthermore, the expression of hepcidin during CAG was mediated by the IL-6/STAT3 pathways. Consequently, the inhibition of hepcidin might be a potential strategy for the prevention of CAG in the future.

## 6. Patents

In the study, we found iron deposition and ferroptosis in specimens of NAG and CAG patents.

## Figures and Tables

**Table 1:** Baseline information of patients diagnosed with NAG and CAG in the study.

	NAG	CAG
Number	20	18
Age range	35–66	41–74
Age mean (±SD)	50.7 ± 11.2	60.39 ± 10.62
Male/female	9/11	9/9
*H. pylori*^+^ (%)	10%	67%
Pathological feature	Mild to moderate chronic non-atrophic gastritis in the antrum	Mild to moderate atrophic gastritis in the antrum

## Data Availability

The data used to support the findings of this study are included within the article.

## Abbreviations

CAG	Chronic atrophic gastritis
GC	Gastric cancer
Dcyt b	Duodenal cytochrome b
DMT1	Divalent metal transporter 1
FPN1	Ferroportin
ROS	Reactive oxygen species
PUFAs	Polyunsaturated fatty acids
GPX4	Glutathione peroxidase 4
MNNG	1-Methyl-3-nitro-1-nitrosoguanidine
4-HNE	4-Hydroxynonenal
FTH	H-ferritin
IL-6	Interleukin-6
TNF-α	Tumor necrosis factor-α
IL-1β	Interleukin-1β
STAT3	Transducer and activator of transcription 3
GAPDH	Glyceraldehyde-3-phosphate dehydrogenase
NAG	Non-atrophic gastritis
Con	Control
TEM	Transmission electron microscope
LIP	Labile iron pool
IBD	Inflammatory bowel disease
NCOA4	Nuclear receptor coactivator 4
LPS	Lipopolysaccharide
IL-6R	IL-6 receptor
JAK2	Janus kinase 2
Th1	T helper 1
IFN-γ	Interferon gamma
